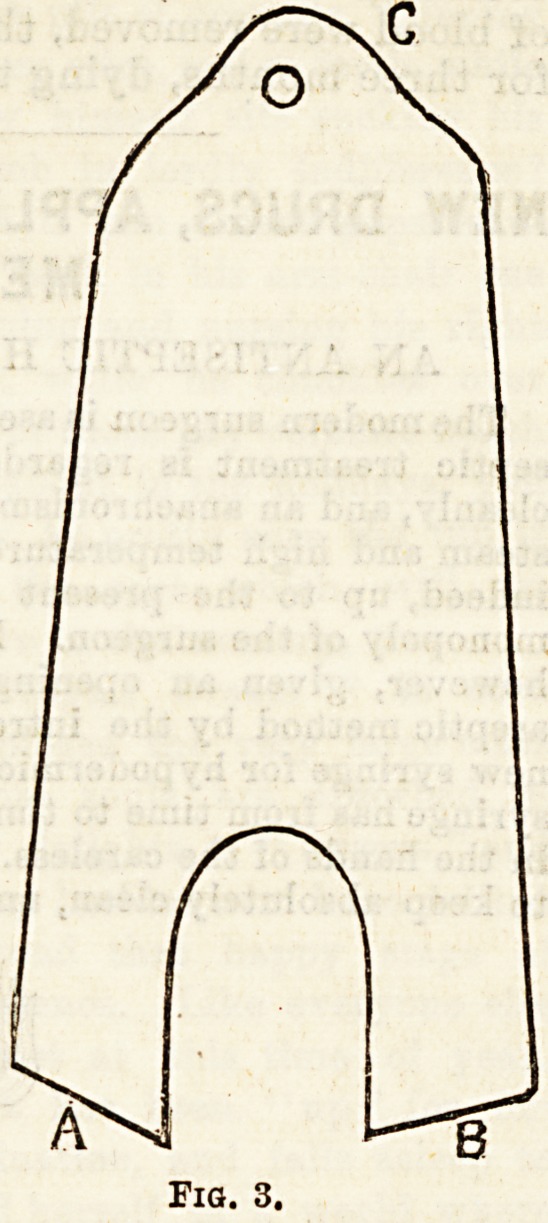# The Treatment of Slighter Forms of In-Knee

**Published:** 1892-12-31

**Authors:** 


### THE BRADFORD INFIRMARY.

### The Treatment of Slighter Forms of In-Knee.

The want of a cheap splint for the treatment of the slighter forms of in-knee resulted in the invention of a simple instrument, which has now been used here with fair success for three years.

*Cases Suitable for Treatment.*—The cases of genu valgum, which have given the best results, are those in which the deformity is due to unequal growth of the lower femoral epiphyses with relaxation of the internal lateral ligament of the knee. Such patients usually show wasting of the leg and thigh muscles, with a tendency to flat foot.

*Description of the Splint.*—The splint consists of two parts, a thigh and leg piece, united by a pivot behind the knee. This allows lateral, but not antero-posterior movement. The thigh piece consists of a hollow trough, which fits the back and sides of the thigh, reaching as high as the fold of the buttock behind, the perineal fold on the inner side and the great trochanter on the outer side. Below the thigh piece is united with the leg piece in the middle line, and prolonged as a flat piece, which lies against the back of the leg trough. On the inner side the thigh piece reaches below the prominent internal condyle, while on the outer side it is cut away to fit the muscular mass on the outer side of the thigh. The leg piece consists of a trough, which fits the back and sides of the leg. It is united above with the thigh piece; below it fits under the back part of the sole of foot, with a hole behind for the heel.

Fig. 1.—The Splint.

*Method of Measuring for the Splint.*—In measuring for the splint a piece of brown paper is fitted round the thigh and cut to fit the thigh. The prolonged piece should reach half way down the back of the leg. It has the shape of the outline sketch Fig. 2. The leg trough is now measured, a pattern being cut in brown paper to fit the back of the child's leg. It has the shape shown in Fig. 3. The two parts are now fitted together. The leg pattern is fitted to the back and sides of the leg, A and B being pinned together under the sole of the foot. The thigh piece is now fitted to the thigh, and the two parts pinned together by a pin passed behind the knee through C in Fig. 3 and C' in Fig. 2. The splints are usually made of tin or thin sheet iron, with a double thickness in the shaded part in Fig. 2. The troughs for the thigh and leg are well padded. The leg and thigh pieces of the splint are rotated until they fit the limb in the deformed position. The splint is applied with the patient lying down, the thigh and leg pieces being fixed with strapping while the limb lies in its deformed position. In genu valgum when the splint is thus applied the prolongation of the thigh piece lies to the inner side of the leg piece. After the splint has been firmly fixed a piece of strapping is fixed to D in Figure 2, and this is drawn outwards until it lies behind the leg piece, where it is firmly fixed with strapping. The limb is thus brought into a straight line. A roller bandage is now applied over the splint. The child is not allowed to walk during the first week. Unless the child is fretful or sleepless the splint is not readjusted for a fortnight. Usually there is no trouble, but in a few cases the heel has been chafed. After the first week the child is allowed to walk with the foot in a boot with a wide heel, or an iron patten is placed beneath the heel. The splint is usually taken off and reapplied once a month. As a general rule, the splint is worn constantly from eight

FIG. 2.—FRONT VIEW OF SPLINT FOR RIGHT THIGH.

FIG. 3.

to ten months. If the case is doing well, it is then applied only during the day, being kept in position by a bandage. The mother removes the splint each night, and rubs the muscles. The child is now taught to strengthen the muscles by flexing and extending the knees, walking along a line, treading on the fore and outer part of the feet. After a month or two the splint may be discarded, the exercises and rubbing being continued. Two splints are worn if both legs are deformed, only one if the defect is unilateral.

In addition to the local treatment cod liver oil is usually given. A simple diet, consisting chiefly of milk, with an egg on every other day, is usually ordered.

Most of the children treated were between five and ten years old, the treatment being unsuited for genu valgum in adolescents. In some cases the splint has failed to correct the deformity, but in many it has been curative, and done away with the necessity for performing osteotomy. The splint is made by Messrs. Arnold, who have kindly allowed the use of their woodcut.

## Figures and Tables

**Fig. 1. f1:**
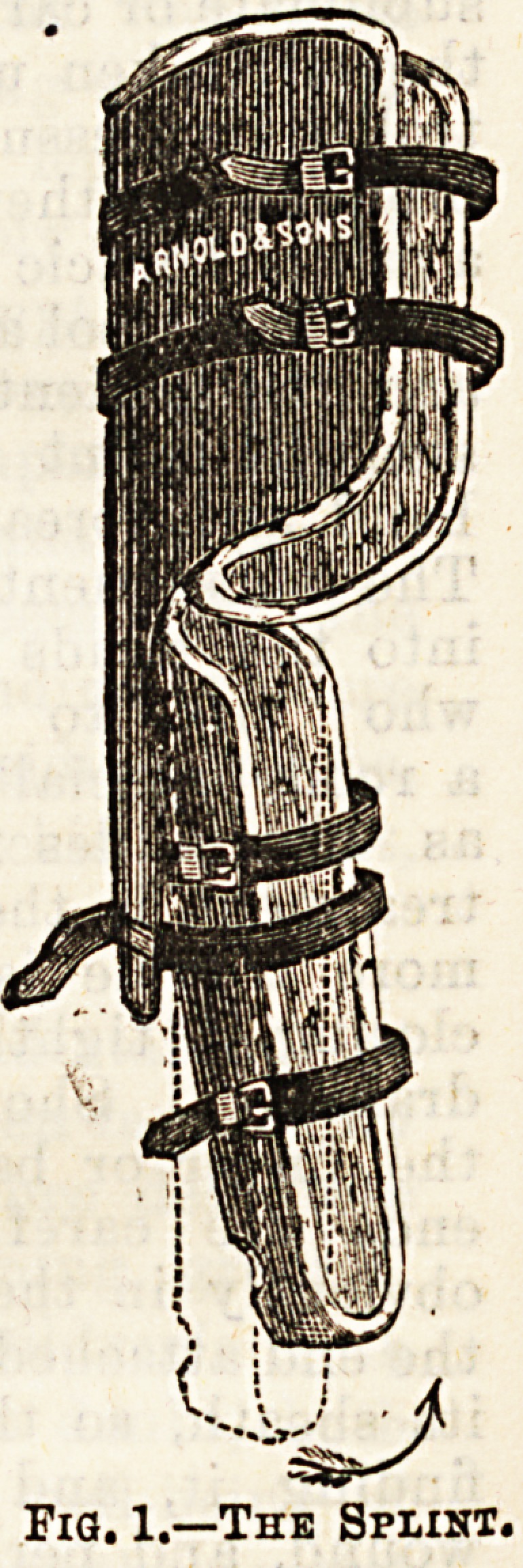


**Fig. 2. f2:**
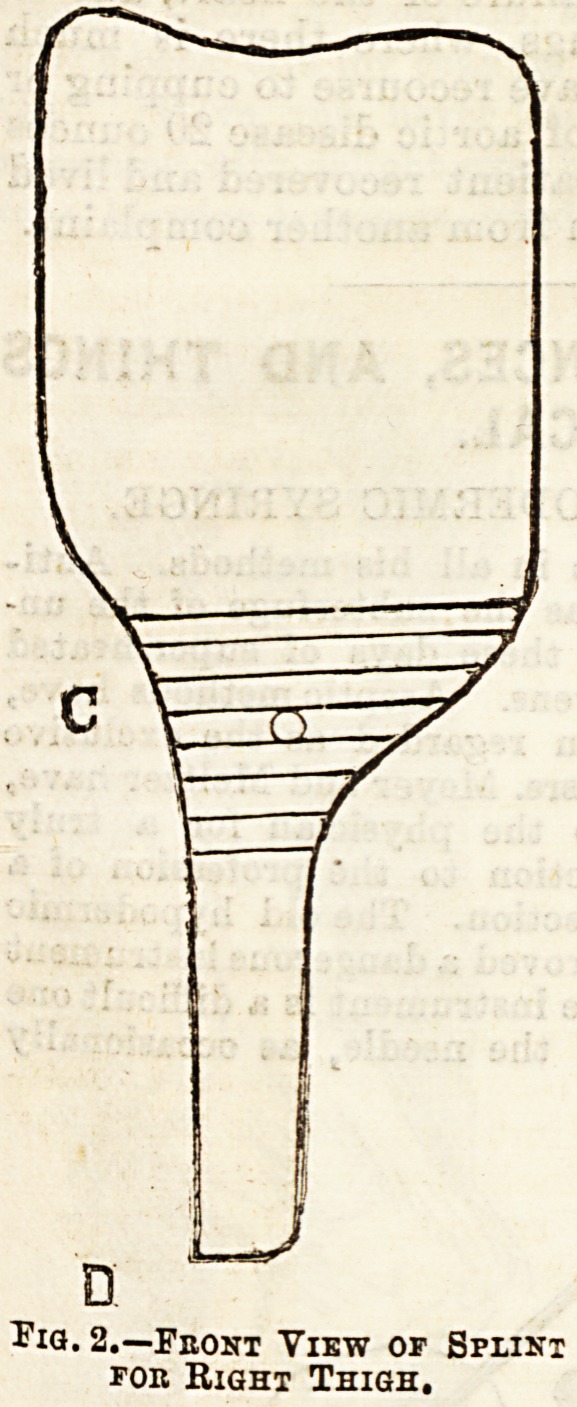


**Fig. 3. f3:**